# Biochemical artifacts in experiments involving repeated biopsies in the same muscle

**DOI:** 10.14814/phy2.286

**Published:** 2014-05-11

**Authors:** Ruud Van Thienen, Gommaar D'Hulst, Louise Deldicque, Peter Hespel

**Affiliations:** 1 Exercise Physiology Research Group – Department of Kinesiology, KU Leuven, Tervuursevest 101, Leuven, B‐3001, Belgium

**Keywords:** Biochemistry, exercise, gene expression, muscle oxygenation, needle biopsy, protein expression, skeletal muscle

## Abstract

Needle biopsies are being extensively used in clinical trials addressing muscular adaptation to exercise and diet. Still, the potential artifacts due to biopsy sampling are often overlooked. Healthy volunteers (*n* = 9) underwent two biopsies through a single skin incision in a *pretest*. Two days later (*posttest*) another biopsy was taken 3 cm proximally and 3 cm distally to the pretest incision. Muscle oxygenation status (tissue oxygenation index [TOI]) was measured by near‐infrared spectroscopy. Biopsy samples were analyzed for 40 key markers (mRNA and protein contents) of myocellular O_2_ sensing, inflammation, cell proliferation, mitochondrial biogenesis, protein synthesis and breakdown, oxidative stress, and energy metabolism. In the pretest, all measurements were identical between proximal and distal biopsies. However, compared with the pretest, TOI in the posttest was reduced in the proximal (−10%, *P* < 0.05), but not in the distal area. Conversely, most inflammatory markers were upregulated at the distal (100–500%, *P* < 0.05), but not at the proximal site. Overall, 29 of the 40 markers measured, equally distributed over all pathways studied, were either up‐ or downregulated by 50–500% (*P* < 0.05). In addition, 19 markers yielded conflicting results between the proximal and distal measurements (*P* < 0.05). This study clearly documents that prior muscle biopsies can cause major disturbances in myocellular signaling pathways in needle biopsies specimens sampled 48 h later. In addition, different biopsy sites within identical experimental conditions yielded conflicting results.

## Abstract (precis)

This study clearly demonstrates that skeletal muscle biopsying per se, at least by causing local tissue inflammation and/or topical deoxygenation, can substantially alter biochemical events happening in needle biopsy specimens sampled at a later day in the same muscle belly. It is crucial to take into account these potential artifacts whenever investigating the cellular mechanisms implicated in adaptation to exercise, recovery, or hypoxia.

## Introduction

The understanding of human muscle physiology and biochemistry during exercise has exponentially grown since Bergström et al. in the early 1960s introduced the use of the percutaneous needle biopsy technique in the context of exercise experiments in healthy volunteers (Bergstrom 1963). For more than 50 years the Bergström needle biopsy procedure has been extensively used to investigate exercise and training effects in muscles. The method also has been improved by the application of suction on the needle cavity to increase sample yield during cutting (Melendez et al. 2007). Often serial biopsies are taken before and after an acute exercise bout and in different experimental conditions, and it is often assumed that the changes measured in the samples obtained, are directly caused by the experimental conditions. Nonetheless, there is clear evidence in literature to prove that needle biopsies cause topical structural damage (Staron et al. 1992), which results in immune‐activation similar to the typical immunological responses seen after muscle damage due to eccentric contractions (Malm et al. 2000). This is also accompanied by a wide spectrum of adaptive biochemical events in the vicinity of the injured muscle area, including regulation of energy substrate metabolism and signaling pathways as well as changes in gene expression (Lundby et al. 2005; Friedmann‐Bette et al. 2012). Thus, studies have demonstrated that biopsies can inhibit muscle ATP and glycogen resynthesis for several days post exercise (Costill et al. 1988; Constantin‐Teodosiu et al. 1996). Furthermore, multiple biopsies within a 2 h time window in m. vastus lateralis markedly increased mRNA contents of interleukin‐6 (IL‐6) and signal transducer and activator of transcription 3 (STAT3), while phosphorylation status of some pivotal signaling proteins in cellular stress, inflammation, and muscle damage was unaltered (Guerra et al. 2011). Vissing et al. (2005) also demonstrated that the expression of several genes that were assumed to be induced by exercise, in fact was an artifact due to the biopsy sampling procedure.

Different factors must be considered when evaluating the potential risk of artifacts in biopsies sampled in the vicinity of another recent biopsy in the same muscle belly. This includes the time interval between the sequential biopsies, the distance between biopsy sites, as well as the orientation of the next biopsy (distal vs. proximal) relative to the previous ones. Surprisingly, the pivotal importance of eliminating biopsy artifacts notwithstanding, work to define optimal conditions for repeated biopsy sampling in the same muscle is only fragmentary. In addition, in most studies published, details on the precise positioning of repeated biopsies are not even mentioned.

We recently planned a series of studies to investigate the effects of exercise training in hypoxia on muscle adaptation, with special attention to downstream targets of intramyocellular O_2_ sensing via HIFs. In this regard it is crucial to know whether results from sequential muscle biopsies reflect the impact of the experimental conditions, indeed, or result from local hypoxia due to biopsy‐induced muscle damage. In fact, needle biopsies could affect local oxygenation status in the muscle by either direct structural damage or postintervention inflammatory responses (Tidball 2005; Smith et al. 2008). In addition, local hypoxia so formed could stimulate calcium release from the sarcoplasmic reticulum and regulate gene transcription of calcium‐calmodulin–dependent intramyocellular proteins such as glucose transporter type 4 (GLUT4), peroxisome proliferator‐activated receptor *γ* coactivator 1‐*α* (PGC‐1α), phospholamban, myoglobin, and several mitochondrial genes (Lewis et al. 1982; Cartee et al. 1991; Eu et al. 2000; Wright et al. 2005; Lanner et al. 2006; Rose et al. 2006, 2009; Deshmukh et al. 2009; Kanatous et al. 2009). Support for such presumptions comes from preliminary studies in our laboratory shown by near‐infrared spectroscopy that a standard biopsy procedure in m. vastus lateralis reduce local muscle oxygenation status for at least a week, with peak deoxygenation values occurring ~48 h post biopsying. On the basis of this observation, we decided to more extensively explore the effects of a needle biopsy procedure in m. vastus lateralis on cellular responses in another biopsy obtained in the same muscle 48 h later. Therefore, we investigated both mRNA and protein contents of key markers in myocellular O_2_ sensing, inflammation, skeletal myogenesis and cell proliferation, mitochondrial biogenesis, oxidative stress, protein synthesis and breakdown, and energy substrate pathways in an experiments involving multiple needle biopsies with a 2‐day interval in m. vastus lateralis.

## Methods

### Subjects

Nine male subjects volunteered to participate in the study after they were informed in detail of the experimental procedures. All subjects were nonsmokers and were diagnosed to be healthy by means of a medical questionnaire. They were instructed not to change their dietary and training habits throughout the study period. Their age and body weight were 21.8 years (range: 21–23) and 62.6 kg (range: 57.8–66.3), respectively. One week before the start of the study the subjects participated in an incremental cycling test (100 + 40 W per 4 min) to determine VO_2_max (62.3 mL min^−1^ kg^−1^; range: 55–74). Furthermore, to allow for valid measurements of muscle oxygenation status by near‐infrared spectroscopy (NIRS) (Ferrari et al. 2004), only subjects with small skinfolds ≤5 mm overlying m. vastus lateralis were included (range: 3.8–5.0 mm). Subjects were also asked not to participate in any strenuous exercise from 2 days prior to the experimental sessions. The study protocol was approved by the local Ethics Committee (KU Leuven) and was in accordance with *The Declaration of Helsinki*. All subjects signed an informed consent.

### Experimental protocol

The subjects participated in a *pretest* and *posttest* session which were interspersed by a 2‐day interval. Each session included measurements of oxygenation status by NIRS and biopsies in m. vastus lateralis using a 5‐mm Bergström needle (see below for details about NIRS and biopsy procedures). At each occasion the subjects reported to the laboratory in the morning between 8:00 and 9:00 am after an overnight fast. On arrival, they rested for 30‐min in a comfortable chair while the skin overlaying the mid part of the m. vastus lateralis belly on the legs was prepared for NIRS measurements (both legs) and biopsies (right leg only). Following the rest period oxygenation status was registered for 10 min where after a standard double muscle biopsy procedure (same incision but different positioning of the needle) in the right leg was performed. Because exercise immediately post biopsying may affect the acute recovery of the wound, and most experiments using muscle biopsies involve exercise post biopsying, subjects cycled for 30 min on a bicycle ergometer (Avantronic^®^ Cyclus 2, Leipzig, Germany) at a workload corresponding to 70% of VO_2_max obtained from the prescreening session. However, no post exercise biopsies were taken to limit the affected zone in the muscle to a single site. The posttest was identical to the pretest, except for the location of the biopsies and omission of the exercise bout following the biopsies.

### NIRS measurements and analysis

**Figure 1.**
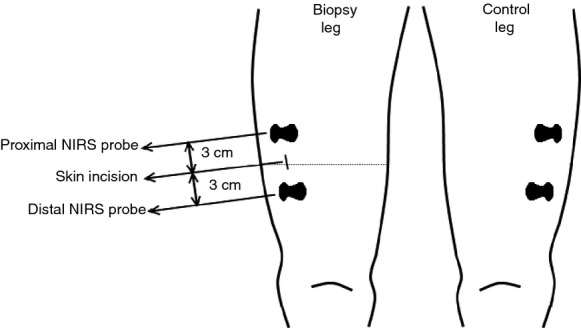
Positioning of the NIRS probes on the biopsy leg and the control leg. Frontal view of the positioning of the NIRS probes relative to the skin incision for muscle biopsying in the right m. vastus lateralis in the pretest. Probe positions were mirrored in the intact left leg which served as a control. The horizontal dotted line indicates the sagittal section as shown in Figure 2. See Methods for further details.

We used the Niro‐200 NIRS instrument (Hamamatsu, Japan) to measure TOI (2 Hz sampling rate). TOI is a valid parameter (Boushel et al. 2001; Quaresima and Ferrari 2009) to assess the fraction of O_2_‐saturated tissue hemoglobin and myoglobin content, reflecting the balance between O_2_ supply and tissue O_2_ consumption. In both the pretest and the posttest two pairs of near‐infrared probes, each consisting of a light emitter and a light detector at 4 cm distance, were fixed on the right m. vastus lateralis (Fig. 1). One pair was positioned 3 cm proximally to the skin incision used in the pretest, whereas the other pair was positioned 3 cm distally. Two other pairs of probes were put identically on the left leg to serve as a control free of biopsy‐induced injury. In the pretest the contour lines of the probes were drawn on the shaved skin to allow for identical repositioning during the posttest.

### Muscle biopsy procedure

**Figure 2.**
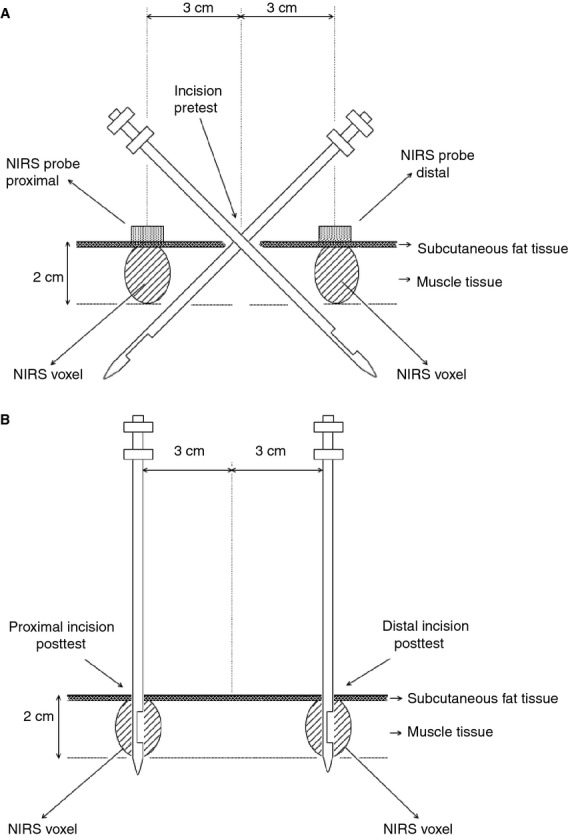
Positioning of the muscle biopsies relative to the skin incision and the appearance of the NIRS voxels in m. vastus lateralis. The picture shows a sagittal section of m. vastus lateralis. The hatched areas indicate the dimensions of the NIRS voxels. Panels A and B show the orientation of the biopsy needle in the pretest and the posttest, respectively. See Methods for further details.

In the pretest right m. vastus lateralis was biopsied using a 5‐mm Bergström‐type needle with suction being applied, through a single 5‐mm incision in the skin under local anesthesia (2% xylocaine without epinephrine, 1 mL subcutaneously) (Fig. 2). The skin incision was made over the belly of the right m. vastus lateralis at 1/3 of the imaginary line connecting the upper lateral border of the patella with the spina iliaca anterior superior. Two biopsies were taken through the same incision, one with the tip of the needle pointing distally from the incision site, and another one with the tip pointing proximally and with an angle of ~45° between the needle and the leg's surface (Fig. 2A). A cm‐scale engraved on the biopsy needles was used as a reference to check adequate positioning of the needle for each biopsy. Each biopsy included the cutting of two samples: after cutting the first sample the needle was rotated 180° around its axis to cut the second sample. Immediately after pressure was applied on the biopsy site until bleeding had completely stopped. The incision was then sutured with adhesive strips (Steri‐Strips^™^, 3M Health Care, Maplewood, MN), and was covered with a plastic gauze (OpSite, Smith & Nephew, London, UK). During the posttest one biopsy was taken 3 cm proximal to the pretest incision, and one biopsy 3 cm distal. These biopsies were taken with the needle inserted 3.5 cm perpendicular to the skin in order to position the cutting window central into the supposed NIRS voxels reaching till 2 cm under the skin (Fig. 2B) (Ferrari et al. 2004).

#### Biochemical analysis – Western blot

Details of the immunoblotting procedures have been described previously (Deldicque et al. 2010; D'Hulst et al. 2013). Briefly, frozen muscle tissue (~20 mg) was homogenized 3 × 5 sec with a Polytronmixer in ice‐cold buffer (1:10, w/v) (50 mmol/L Tris‐HCl pH 7.0, 270 mmol/L sucrose, 5 mmol/L EGTA, 1 mmol/L EDTA, 1 mmol/L sodium orthovanadate, 50 mmol/L glycerophosphate, 5 mmol/L sodium pyrophosphate, 50 mmol/L sodium fluoride, 1 mmol/L DTT, 0.1% Triton‐X 100 and a complete protease inhibitor tablet [Roche Applied Science, Vilvoorde, Belgium]). Homogenates were then centrifuged at 10,000*g* for 10 min at 4°C. The supernatant was collected and immediately stored at −80°C. The protein concentration was measured using the DC protein assay kid (Bio‐Rad laboratories, Nazareth, Belgium). Using SDS‐PAGE (8–12% gels), 30–80 μg of proteins were separated and transferred to PVDF membranes. Subsequently, membranes were blocked with TBST (tris‐buffered saline, 0.1% Tween 20) containing 5% nonfat dry milk for 1 h and afterwards incubated overnight (4°C) with the following antibodies (1:1000, Cell Signaling, Leiden, the Netherlands): total eukaryotic elongation factor 2 (eEF2), hypoxia‐inducible factor 1 *α* (HIF‐1α), neuronal nitric oxide synthase (nNOS), phospho‐ribosomal protein S6 kinase 1 (S6K1) Thr389, total S6K1, phospho‐eukaryotic initiation factor 2 α (eIF2*α*) Ser51, total eIF2*α*, phospho‐AMP‐activated protein kinase (AMPK) Thr172, total AMPK, phospho‐glycogen synthase kinase 3*β* (GSK‐3*β*) Ser9, nuclear factor kappa B inhibitor *α* (I*κ*B‐*α*), pan Akt, phospho‐Akt Ser473. Horseradish peroxidase‐conjugated anti‐mouse (1:10,000), anti‐rabbit (1:5000), or anti‐goat (1:20,000) secondary antibodies (Sigma‐Aldrich, Bornem, Belgium) were used for chemiluminescent detection of proteins. Membranes were scanned and quantified with Genesnap and Genetools softwares (Syngene, Cambridge, UK), respectively. Then, membranes were stripped and reprobed with the antibody for the total form of the respective protein to ascertain the relative amount of the phosphorylated protein compared to the total form throughout the whole experiment. The results are presented as the ratio protein of interest/eEF2 or as the ratio phosphorylated/total form of the proteins when the phosphorylation status of the protein was measured. eEF2 protein was not different between pre‐ and posttest. A value of 1.0 was assigned to the mean value of the samples from the pretest from the proximal as well as from the distal site to which the other corresponding value from the posttest was reported.

#### RNA extraction and reverse transcription

The method used for reverse transcription is described in detail elsewhere (Vincent et al. 1985; Jamart et al. 2011). Briefly, total RNA was extracted using TRIzol (Invitrogen, Vilvoorde, Belgium) from 20 to 25 mg of frozen muscle tissue. RNA quality and quantity were assessed by spectrophotometry with a Nanodrop (Thermo Scientific, Erembodegem, Belgium). One microgram of RNA was reverse transcribed using the High Capacity cDNA Reverse Transcription kit (Applied Biosystems, Gent, Belgium) according to manufacturer's instructions.

#### Real‐time qPCR analysis

A SYBR Green‐based master mix (Applied Biosystems) was used for real‐time PCR analyses using the ABI PRISM 7300 (Applied Biosystems). Real‐time PCR primers were designed for HIF‐1*α* and HIF‐2*α*, vascular endothelial growth factor (VEGF), regulated in development and DNA damage responses protein 1 (REDD‐1), nNOS, tumor necrosis factor *α* (TNF‐*α*), interleukin 6 (IL‐6), cyclophilin A (CycloA), IKB‐*α*, myoblast determination protein 1 (MyoD), myogenic factor 5 (Myf5), myogenic regulatory factor 4 (MRF‐4), myogenin, proliferating cell nuclear antigen (PCNA), peroxisome proliferator‐activated receptor *γ* coactivator 1‐*α* (PGC‐1*α*), mitochondrial transcription factor A (TFAM), peroxisome proliferator‐activated receptor *γ* (PPAR‐*γ*), ubiquitin ligase Atrogin1/Muscle Atrophy F‐box (MAFBx), muscle RING‐finger protein‐1 (MURF‐1), Bcl‐2/adenovirus E1B 19 kDa protein‐interacting protein 3 (BNIP3), superoxide dismutase 1 and 2 (SOD‐1 and SOD‐2), catalase, nicotinamide adenine dinucleotide phosphate‐oxidase (NAPDH‐ox), glucose transporter type 4 (GLUT‐4), S6K1, Akt‐1, Akt‐2, GSK‐3α and GSK‐3β, and AMPK‐α1 and AMPK‐α2. Thermal cycling conditions consisted of 40 three‐step cycles including denaturation of 30 sec at 95°C, annealing of 30 sec at 58°C and extension of 30 sec at 72°C. All reactions were performed in triplicate. To compensate for variations in input RNA amounts and efficiency of reverse transcription, glyceraldehyde‐3‐phosphate dehydrogenase (GAPDH), ribosomal proteins L4 and L19 (RPL4 – RPL19) and *β*‐2‐microglobulin (*β*‐2‐MG) mRNA were quantified, and results were normalized to these values. These genes were chosen out of five normalization genes using the GeNorm applet according to the guidelines and theoretical framework described elsewhere (Vandesompele et al. 2002). A value of 1.0 was assigned to the mean value of the samples from the pretest from the proximal as well as from the distal site to which the other corresponding value from the posttest was reported.

### Statistical analysis

Prior to statistical analysis, NIRS‐data were preprocessed with a Butterworth filter in customer level made mathematical software (Matlab, The Mathworks, Natick, MA). NIRS outputs were first visually inspected to evaluate whether TOI‐values had acquired a steady‐state. For each of the four measurement sites a single average TOI‐value was calculated. The effects of the muscle biopsy procedure on TOI and biochemical measurements were evaluated using a repeated‐measures analysis of variance (ANOVA) (Statistica 9.0, Statsoft, Tulsa, OK). A two‐way ANOVA was performed to assess the main effects of biopsy location (proximal vs. distal), and time (pretest vs. posttest). Bonferroni post hoc comparisons were used when appropriate. A probability level (*P*) ≤ 0.05 was considered statistically significant. All data are expressed as means ± SEM.

## Results

### Muscle tissue oxygenation index

**Figure 3.**
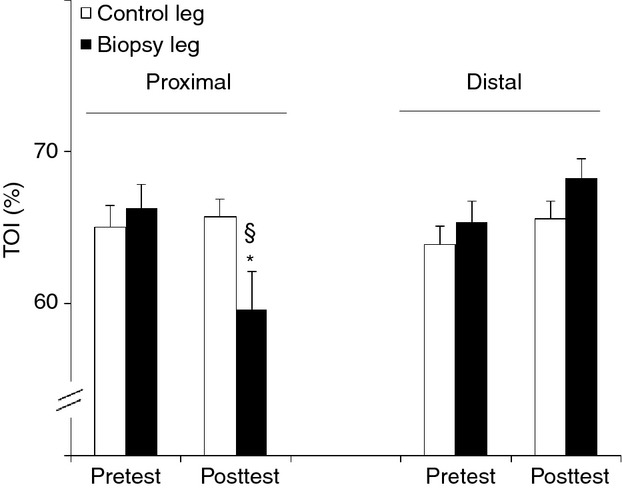
Effect of needle biopsying on muscle oxygenation status. Values are means ± SEM (*n* = 9) and represent muscle tissue oxygenation index (TOI, %) measured by NIRS in the pretest and in the posttest. Biopsies were taken as shown in Figure 2. A standard biopsy procedure was performed in m. vastus lateralis while TOI was measured by near‐infrared spectroscopy 3 cm proximal and 3 cm distal to the pretest incision site. The contralateral leg served as a control leg. See Methods for further details. *P < 0.05 compared with control leg. ^§^*P* < 0.05 compared with pretest.

Tissue oxygenation index (TOI) was measured proximally and distally (see Fig. 2A) in both legs before (pretest) and 48 h after (posttest) the pretest biopsies (Fig. 3). TOIs were identical between legs in the pretest. However, in the posttest, compared with the control leg TOI in the biopsied leg was reduced by ~10% at the proximal site (*P* < 0.05), but not at the distal site.

### Muscle mRNA and protein contents

**Figure 4.**
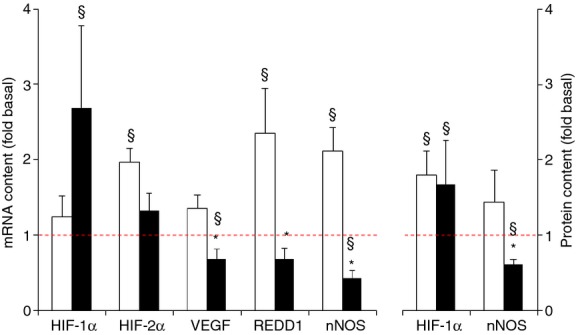
Effect of needle biopsying on key markers in myocellular oxygen sensing. Data are means ± SEM (*n* = 9) and are expressed as fold increase relative to the baseline obtained from the pretest biopsies. mRNA (left panel) and protein content (right panel) were measured in muscle tissue sampled by needle biopsy either 3 cm proximal (open bars) or 3 cm distal (filled bars) to the pretest incision. See Methods for further details. *P < 0.05 compared with proximal, ^§^*P* < 0.05 compared with pretest.

**Figure 5.**
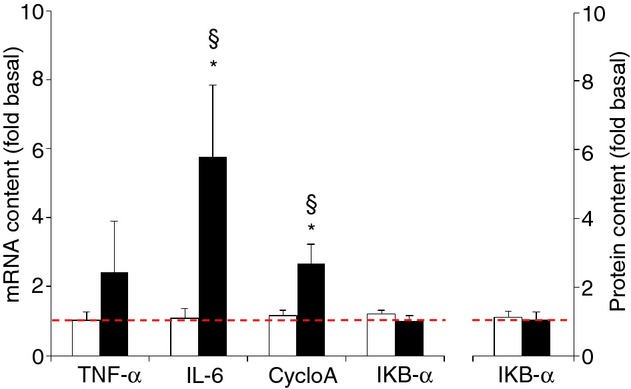
Effect of needle biopsying on key markers in myocellular inflammation. Data are means ± SEM (*n* = 9) and are expressed as fold increase relative to the baseline obtained from the pretest biopsies. mRNA (left panel) and protein content (right panel) were measured in muscle tissue sampled by needle biopsy either 3 cm proximal (open bars) or 3 cm distal (filled bars) to the pretest incision. See Methods for further details. *P < 0.05 compared with proximal, ^§^*P* < 0.05 compared with pretest.

**Figure 6.**
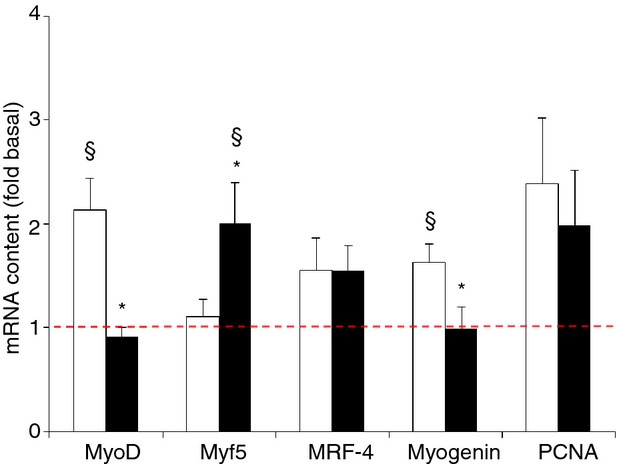
Effect of needle biopsying on key markers in skeletal myogenesis and cell proliferation. Data are means ± SEM (*n* = 9) and are expressed as fold increase relative to the baseline obtained from the pretest biopsies. mRNA content was measured in muscle tissue sampled by needle biopsy either 3 cm proximal (open bars) or 3 cm distal (filled bars) to the pretest incision. See Methods for further details. *P < 0.05 compared with proximal, ^§^*P* < 0.05 compared with pretest.

**Figure 7.**
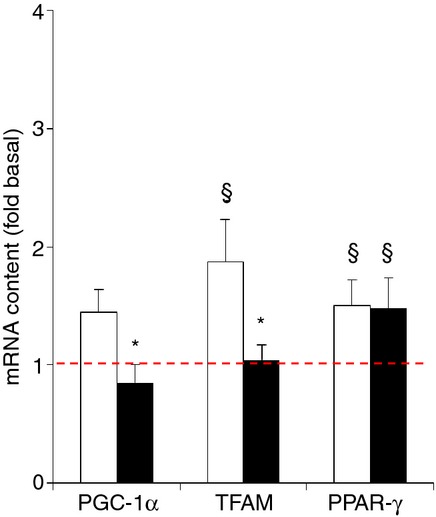
Effect of needle biopsying on key markers in mitochondrial biogenesis and regulation. Data are means ± SEM (*n* = 9) and are expressed as fold increase relative to the baseline obtained from the pretest biopsies. mRNA content was measured in muscle tissue sampled by needle biopsy either 3 cm proximal (open bars) or 3 cm distal (filled bars) to the pretest incision. See Methods for further details. *P < 0.05 compared with proximal, ^§^*P* < 0.05 compared with pretest.

**Figure 8.**
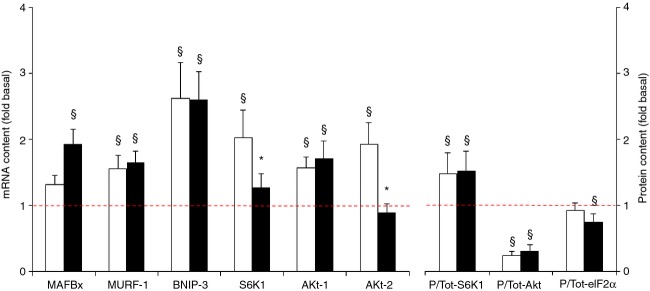
Effect of needle biopsying on key markers in protein synthesis and breakdown. Data are means ± SEM (*n* = 9) and are expressed as fold increase relative to the baseline obtained from the pretest biopsies. mRNA (left panel) and protein phosphorylation status (S6K1, Akt, and eIF2α) (right panel) were measured in muscle tissue sampled by needle biopsy either 3 cm proximal (open bars) or 3 cm distal (filled bars) to the pretest incision. See Methods for further details. *P < 0.05 compared with proximal, ^§^*P* < 0.05 compared with pretest.

**Figure 9.**
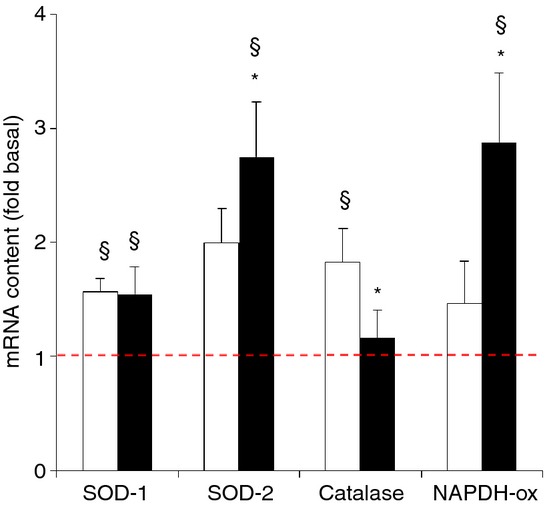
Effect of needle biopsying on key markers in oxidative stress. Data are means ± SEM (*n* = 9) and are expressed as fold increase relative to the baseline obtained from the pretest biopsies. mRNA content was measured in muscle tissue sampled by needle biopsy either 3 cm proximal (open bars) or 3 cm distal (filled bars) to the pretest incision. See Methods for further details. *P < 0.05 compared with proximal, ^§^*P* < 0.05 compared with pretest.

**Figure 10.**
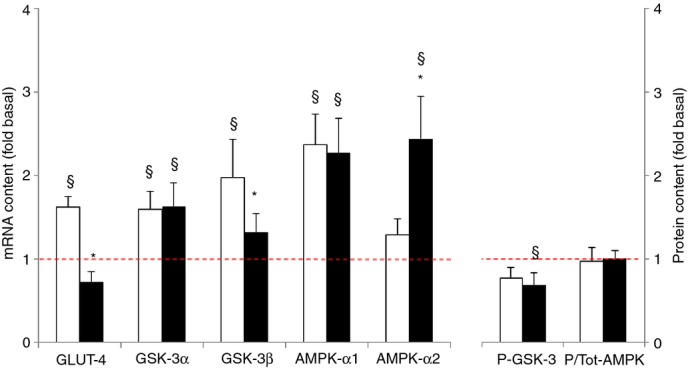
Effect of needle biopsying on key markers in glucose and lipid metabolism. Data are means ± SEM (*n* = 9) and are expressed as fold increase relative to the baseline obtained from the pretest biopsies. mRNA (left panel) and protein phosphorylation status (AMPK) or phosphorylated protein content (GSK‐3) (right panel) were measured in muscle tissue sampled by needle biopsy either 3 cm proximal (open bars) or 3 cm distal (filled bars) to the pretest incision. See Methods for further details. *P < 0.05 compared with proximal, ^§^*P* < 0.05 compared with pretest.

Biochemical assays were performed in the proximal and distal biopsy samples obtained in both the pretest and the posttest (see Fig. 2). Proximal and distal samples in the pretest yielded identical results for all measurements (Table 1). Therefore, posttest data are expressed relative to the corresponding pretest value (Figs. 4–10).

**Table 1.** *P*‐values for comparison of proximal and distal biopsies in the pretest.

	*P*‐value		*P*‐value
Oxygen sensing pathways		Protein synthesis and breakdown markers	
HIF‐1α	0.78	MAFBx	0.99
HIF‐2α	0.53	MURF‐1	0.94
VEGF	0.91	BNIP‐3	0.97
REDD1	0.99	S6K1	0.99
nNOS	0.51	Akt‐1	0.83
HIF‐1α protein content	0.99	Akt‐2	0.90
nNOS protein content	0.99	P/Tot S6K1 protein content	0.99
		P/Tot Akt protein content	0.99
Inflammation markers		P/Tot elF2α protein content	0.99
TNF‐α	0.96		
IL‐6	0.97	Oxidative stress markers	
CycloA	0.99	SOD‐1	0.72
IKB‐α	0.64	SOD‐2	0.78
IKB‐α protein content	0.99	Catalase	0.88
		NAPDH‐oxidase	0.87
Myogenesis and cell proliferation markers			
MyoD	0.99	Glucose and lipid metabolism markers	
Mif5	0.86	GLUT‐4	0.80
MRF‐4	0.97	GSK‐3α	0.82
Myogenin	0.75	GSK‐3β	0.72
PCNA	0.98	AMPK‐α1	0.78
		AMPK‐α2	0.99
Mitochondrial biogenesis markers		P‐GSK‐3 protein content	0.99
PGC‐1α	0.88	P/Tot AMPK protein content	0.99
TFAM	0.99		
PPAR‐Y	0.97		

A two‐way repeated‐measures analysis of variance was performed to assess main effects of biopsy position (proximal vs. distal biopsy) and time (pretest vs. posttest). Only the *P*‐values for the comparison of proximal versus distal biopsies in the pretest are reported in this table. Data shown refer to the muscle mRNA contents (unless stated “protein content”) of all myocellular metabolism markers described in the Methods and Results section.

#### Oxygen sensing pathways

Compared with the pretest, mRNA contents of HIF‐1α, HIF‐2α, REDD1, and nNOS were upregulated in the posttest (*P* < 0.05) (Fig. 4). However, HIF‐1α mRNA content was increased in the distal biopsy only (+169%), while HIF‐2α (+97%), REDD1 (+135%), and nNOS (+112%) were increased only proximally. VEGF (−32%) and nNOS (−58%) mRNA content was downregulated in the posttest, but only in the distal biopsy (*P* < 0.05). mRNA contents in the posttest were significantly different between proximal and distal biopsies for VEGF, REDD1, and nNOS (*P* < 0.05). Compared with the pretest, HIF‐1α protein content in the posttest was elevated at both the proximal and distal site, whereas mRNA content was increased only distally (*P* < 0.05). For nNOS though both protein and mRNA expressions were lower in the distal biopsy than in the proximal one (*P* < 0.05).

#### Inflammation markers

Compared with the pretest, mRNA contents of IL‐6 (+476%), CycloA (+168%), and TNF‐α (+141%, *P* < 0.10) were increased at the distal biopsy site (*P* < 0.05), but not proximally (Fig. 5). mRNA contents in the posttest were different between proximal and distal biopsies for both IL‐6 and CycloA (*P* < 0.05). IKB‐α expression was similar between the pretest and the posttest both at the mRNA level and at the protein level.

#### Myogenesis and cell proliferation markers

In the posttest mRNA contents of MyoD (+113%), Myf5 (+100%), and Myogenin (+63%) were higher than in the pretest (*P* < 0.05) (Fig. 6), while MRF‐4 content was unchanged. Nonetheless, MyoD and Myogenin mRNAs were increased only proximally, whereas Myf5 was elevated at only the distal site (*P* < 0.05). mRNA contents of MyoD, Myf5, and Myogenin were significantly different between the proximal and distal biopsies (*P* < 0.05).

#### Mitochondrial biogenesis markers

Compared with the pretest, mRNA contents of TFAM (+87%) and PPAR‐γ (+50%) were elevated in the posttest, but for TFAM this only occurred in the proximal biopsy (*P* < 0.05) and not in the distal one (Fig. 7). PGC‐1α content significantly changed at neither biopsy site although a trend toward an increased content at the proximal biopsy site was observed (*P* = 0.10). mRNA contents were different between the distal and proximal biopsies for both PGC‐1α and TFAM.

#### Protein synthesis and breakdown markers

mRNA contents of MURF‐1 (approximately +60%), BNIP‐3 (approximately +160%), and Akt‐1 (approximately +65%) were increased in the posttest in both the distal and proximal biopsies (*P* < 0.05) (Fig. 8). mRNA level of S6K1 (+103%) and Akt‐2 (+93%) was upregulated only proximally, whereas MAFBx mRNA content was upregulated only distally (+94%) (*P* < 0.05). For S6K1 and Akt‐2 significant differences in mRNA contents were found between the proximal and distal biopsies. There was no good match between changes in total protein and mRNA contents. In fact, total protein contents of S6K1, Akt, and elF2α were constant between the pretest and the posttest (data not shown). Conversely, the phosphorylated fraction of Akt was consistently decreased in the posttest (approximately −70%), S6K1, on the other hand, was increased (~50%) (*P* < 0.05). The fraction of phosphorylated elF2α was similar between the pretest and the posttest.

#### Oxidative stress markers

Compared with the pretest all markers of oxidative stress measured were increased, at least in one of the two sites studied (*P* < 0.05) (Fig. 9). Only SOD‐1 mRNA (approximately +55%) upregulation was similar between biopsy sites, while SOD‐2 (+175%) and NAPDH‐oxidase (+188%) mRNA contents were increased only in the distal biopsy samples. In contrast, catalase mRNA level (+83%) was elevated only proximally. mRNA values were significantly different between distal and proximal muscle samples for SOD‐2, catalase, as well as NAPDH‐oxidase (*P* < 0.05).

#### Glucose and lipid metabolism markers

All investigated mRNA's encoding glucose and lipid metabolism markers were higher in the posttest than in the pretest (*P* < 0.05) (Fig. 10). However, GSK3‐α (approximately +60%) and AMPK‐α1 (approximately +130%) mRNAs were higher in both distal and proximal samples, GLUT‐4 (+62%) and GSK‐3β (+98%) mRNAs on the other hand, were increased only at the proximal biopsy site (*P* < 0.05). Conversely, AMPK‐α2 (+144%) showed upregulation only distally (*P* < 0.05). There was no good match between changes in total protein and mRNA contents. In fact total protein content of AMPK was constant between the pretest and the posttest (data not shown). Furthermore, a minor decline occurred in the phosphorylated form of GSK3 protein at the distal biopsy site (*P* < 0.05). The fraction of phosphorylated AMPK was similar between the pretest and the posttest.

## Discussion

Needle biopsying to obtain skeletal muscle tissue in healthy volunteers has become a standard procedure in exercise physiology and biochemistry research. In fact, current knowledge on myocellular adaptation to exercise and recovery largely originates from studies using the Bergström needle biopsy procedure (Bergstrom 1963, 1975). Nonetheless, it is well documented that needle biopsies per se cause topical structural damage and inflammation (Staron et al. 1992; Malm et al. 2000), which conceivably may affect observations in muscle tissue sampled in the vicinity of other recent biopsies. However, literature data on the confounding effects of biopsying per se on biochemical events in muscle are rather fragmentary though (Costill et al. 1988; Constantin‐Teodosiu et al. 1996; Malm et al. 2000; Lundby et al. 2005; Vissing et al. 2005; Guerra et al. 2011; Friedmann‐Bette et al. 2012). Therefore, in this study we took muscle samples from m. vastus lateralis using a conventional 5 mm Bergström‐type biopsy needle. In the pretest a muscle sample was cut both 3 cm proximal and 3 cm distal to a central skin incision (see Fig. 2). Forty‐eight hours later in the posttest, additional samples were taken adjacent to the earlier proximal and distal biopsy sites. We compared mRNA and protein contents of a series of key markers for myocellular O_2_ sensing, inflammation, cell proliferation, mitochondrial biogenesis, protein synthesis and breakdown, oxidative stress, and energy metabolism between the pretest and the posttest samples. In the pretest, all measurements yielded identical results for samples obtained either proximally or distally from the skin incision. However, the pretest caused major alterations in all signaling pathways assessed in the posttest biopsies. Twenty‐nine of the 40 cellular markers measured were either up‐ or downregulated by 50–500%. However, proximal and distal samples yielded discrepant results for about half of these markers, with mRNA data being much more volatile than measurements of protein expression and phosphorylation status.

Independent of the etiology, acute muscle injury causes fiber damage and necrosis. This ignites an inflammatory reaction that is an essential step toward regeneration. Inflammatory cells produce several growth factors as well as stimulate the release of muscle regeneratory factors needed for muscle progenitor cell activation and differentiation (Tidball 1995, 2005; Huard et al. 2002; Jarvinen et al. 2005; Turner and Badylak 2012). Accordingly, it was shown that the microtrauma caused by a biopsy procedure elicits a local inflammatory response (Costill et al. 1988; Tidball 2005; Smith et al. 2008) which is full‐blown within 24–48 h. In this study, compared with the pretest all mRNA markers of inflammation, that is IL‐6, TNF‐α, and CycloA, but not IκB‐α (see Fig. 5), were markedly increased 48 h after the pretest biopsies (approximately posttest). The fivefold increase in IL‐6 mRNA was most explicit, which is in line with previous findings (Guerra et al. 2011). However, inflammatory markers were increased only in the distal biopsies, not in the proximal ones. A likely explanation for such differential response between samples is the formation of a hematoma “downstream” to the biopsy area. During the exercise bout following the pretest biopsy, blood and capillary filtrate due to gravitation conceivably drained from proximal to distal, which in turn triggered an inflammatory response at the distal site (Tidball 1995; Jarvinen et al. 2005). Following each biopsy we applied local compression for 5–10 min to stop visible bleeding before the subjects returned to the upright position to perform the exercise bout. However, such procedure seemingly is inadequate to fully eliminate internal bleeding during subsequent muscle contractions. The absence of activation of inflammatory markers at the proximal site also proves that the inflammation certainly was not due to the exercise per se but specifically to the biopsy procedure.

We also postulated that mechanical damage to the microcirculation due to needle insertion, in conjunction with the ongoing inflammatory processes, might affect local oxygenation status and thereby impact on O_2_‐sensing pathways. Therefore, we measured local tissue oxygenation status (TOI) by near‐infrared spectroscopy. Baseline TOIs in the pretest were identical between the two legs. In the control leg TOIs yielded normal basal values also in the posttest (Quaresima et al. 2002; Tew et al. 2010). However, in the biopsied leg TOI dropped by approximately 10–15% in the posttest (see Fig. 3), yet only at the proximal site. Therefore, contrary to our hypothesis, ongoing inflammation processes do not seem to affect local oxygenation status as inflammatory markers were only upregulated at the distal site, whereas TOI was only decreased at the proximal site. Although the origin of local muscle deoxygenation could not be determined at the hand of our data, the decrease in TOI could be the trigger for elevated HIF‐2α, REDD1, and nNOS mRNA contents at the proximal site. In contrast, in the distal but not in the proximal biopsies VEGF, REDD1, and nNOS mRNAs were slightly decreased, while HIF‐1α mRNA as well as protein content were increased (Fig. 4). In fact, only for HIF‐1α protein expression proximal and distal biopsies showed an identical approximately twofold increment, confirming that HIF‐1 stabilization is not only dependent on the level of hypoxia (Zhong et al. 2010; Luo et al. 2011) as those results do not match TOI data perfectly. Also, changes in HIF‐1α protein content did not match changes in mRNA content, which has already been shown after resistance exercise (Ameln et al. 2005).

It is well established that mitochondria play an important role in hypoxia adaptation by regulation of cellular energy balance and reactive oxygen species homeostasis in relation with HIF‐1 stabilization (Solaini et al. 2010). PGC‐1α, TFAM, and PPAR‐γ are implicated in mitochondrial biogenesis and function (Scarpulla 1813; Jornayvaz and Shulman 2010; Wenz 2013). Concomitant with the drop of TOI at the proximal site in the posttest, mRNA levels of TFAM and PPAR‐γ were elevated, while PGC‐1α mRNA tended to increase (see Fig. 7). However, once again contrasting results were found between proximal and distal biopsies for both PGC‐1 and TFAM mRNAs. In fact, only PPAR‐γ mRNA content was increased to the same degree in muscle tissue sampled either proximally or distally. Interpretation of the aforementioned results also requires considering the precise positioning as well as the frequency of the repeated biopsies (see Figs. 1 and 2). In the pretest the samples were cut approximately 2–3 cm below the bottom end of the virtual NIRS voxel with the express purpose to avoid structural muscle damage within the NIRS voxel, which would invalidate the measurements. Conversely, in the posttest, samples were cut within the NIRS voxel. The biopsies in the posttest thus reflect cellular and molecular events happening in intact muscle tissue within 2–3 cm distance from another biopsy taken 48 h before. We limited the number of biopsies in the pretest to just two, yet still observed major impact on the posttest measurements. It is reasonable to assume that the effects of muscle biopsying on local oxygenation status and cellular O_2_ sensing probably will be exaggerated if even more than two needle biopsies were taken from the same muscle within one experiment.

Muscle damage by needle biopsying probably also results in increased protein turnover by activation of both catalytic and anabolic pathways (Huard et al. 2002; Watford 2003; Butterfield et al. 2006). At the catalytic side of protein turnover, in the posttest mRNA levels of the ubiquitin‐ligases MAFBx and MURF‐1, as well as mRNA of BNIP‐3, which is implicated in cellular autophagy and apoptosis (Zhang and Ney 2009), were increased in both the proximal and distal biopsies (see Fig. 8). Conversely, at the anabolic side of protein turnover, phosphorylation of Akt and its downstream target GSK‐3 was decreased in the posttest, whereas phosphorylation of S6K1 was increased, indicating that additional regulatory signals acted on S6K1 itself or between Akt and S6K1, probably at the level of mTOR, the kinase for S6K1 at Thr389. Potential candidates could have been AMPK and REDD1 as the latters are known to inhibit mTOR (Brugarolas et al. 2004; Liu et al. 2006). In the present case, a decrease in AMPK phosphorylation or in REDD1 content could have explained the increase in S6K1 phosphorylation but AMPK phosphorylation was unchanged in the posttest and REDD1 mRNA level increased at the proximal site while not changing at the distal site. Therefore, another untested mechanism should have led to the increased S6K1 in the posttest. Importantly, changes in S6K1, Akt GSK‐3, AMPK, and elF2α phosphorylation were consistent between tissue sampled either proximally or distally in the muscle. This was not the case for most of the changes in mRNA levels. Compared with the pretest AMPK‐α1 and α2 mRNAs were substantially upregulated in the posttest, yet for AMPK‐α2 this occurred only in the distal biopsies. The same holds true for MyoD, Myf5, and myogenin mRNAs, nuclear transcription factors that play an important role in muscle repair (Zanou and Gailly 2013), which also exhibited discrepant responses between proximal and distal biopsies (see Fig. 6). In addition, for none of the above variables the changes in mRNA content between the pretest and the posttest translated into similar changes in the total protein content of the corresponding proteins.

It is also well known that regulation of redox balance by the NADPH‐oxidase, SOD, and catalase enzymes plays an important role in regulation of wound healing post injury (Soneja et al. 2005; Filippin et al. 2009). Accordingly, compared with the pretest both SOD‐1, SOD‐2, catalase, and NADPH‐oxidase mRNA levels were increased. However again, only for SOD‐1 similar changes were found between the proximal and the distal biopsies. It is noteworthy to mention that by analogy with the mRNA markers of inflammation (TNF‐α, IL‐6, and CycloA, see Fig. 5), also NADPH‐oxidase mRNA content was increased only distally in the muscle belly. Indeed, both leukocyte and muscle cell NADPH‐oxidases play a pivotal role in tissue inflammation and repair by producing the reactive oxygen species superoxide and as such contribute to myocellular oxidative stress and regulate proliferation of skeletal muscle precursor cells (Bokoch and Knaus 2003; Mofarrahi et al. 2008; Jiang et al. 2011).

An important message from the present work is that the exact site of sample cutting within the muscle belly, rather than the location of the skin incision, is the reference to use whenever planning repeated muscle biopsies in the same muscle with only days in between. In fact in most studies involving percutaneous needle biopsies two muscle samples are cut via a single skin incision. Typically one sample is cut with the needle pushed up 2–3 cm proximally into the muscle belly, while the second tissue sample is cut 2–3 cm distally to the incision. Using a similar procedure for later follow‐up biopsies via a new skin incision 3–5 cm either distally or proximally to the first incision, does not exclude to cut samples within a 2 cm radius from an earlier biopsy spot.

In conclusion, this study clearly demonstrates that needle biopsies per se, at least by causing local tissue inflammation and/or topical deoxygenation, can substantially alter biochemical events happening in needle biopsy specimens sampled at a later day in the same muscle belly. It is crucial to take into account these potential artifacts whenever investigating the cellular mechanisms implicated in adaptation to exercise, recovery, or hypoxia. mRNA data clearly are much more sensitive to biopsy‐induced artifacts than protein measurements. We recommend that the methodology section of studies involving repeated muscle biopsies from the same muscle with only a few days in between, provides a detailed description of the precise tissue sampling sites within the muscle, rather than just mentioning the distance between skin incisions which does not exclude artifacts at all.

## Acknowledgments

The authors thank all subjects for participating in this study.

## Conflict of Interest

None declared.
